# Human Upf1 is a highly processive RNA helicase and translocase with RNP remodelling activities

**DOI:** 10.1038/ncomms8581

**Published:** 2015-07-03

**Authors:** Francesca Fiorini, Debjani Bagchi, Hervé Le Hir, Vincent Croquette

**Affiliations:** 1 Institut de Biologie de l'Ecole Normale Supérieure, CNRS UMR8197, Paris 75230, France; 2 Institut de Biologie de l'Ecole Normale Supérieure, INSERM U1024, Paris 75230, France; 3 Laboratoire de Physique Statistique, Ecole Normale Supérieure, Université Pierre et Marie Curie Paris, Université Paris Diderot, CNRS, 24 rue Lhomond, Paris 75005, France

## Abstract

RNA helicases are implicated in most cellular RNA-dependent events. In eukaryotes however, only few have been functionally characterized. Upf1 is a RNA helicase essential for nonsense-mediated mRNA decay (NMD). Here, using magnetic tweezers and bulk assays, we observe that human Upf1 is able to translocate slowly over long single-stranded nucleic acids with a processivity >10 kb. Upf1 efficiently translocates through double-stranded structures and protein-bound sequences, demonstrating that Upf1 is an efficient ribonucleoprotein complex remodeler. Our observation of processive unwinding by an eukaryotic RNA helicase reveals that Upf1, once recruited onto NMD mRNA targets, can scan the entire transcript to irreversibly remodel the mRNP, facilitating its degradation by the NMD machinery.

## Abstract (web summary)

Upf1 is a multifunctional helicase involved in various DNA- and RNA-related processes, including nonsense-mediated mRNA decay (NMD). Here the authors demonstrate that Upf1 is a highly processive ribonucleoprotein complex remodeler—a capability likely important for Upf1's NMD function.

## Graphical abstract

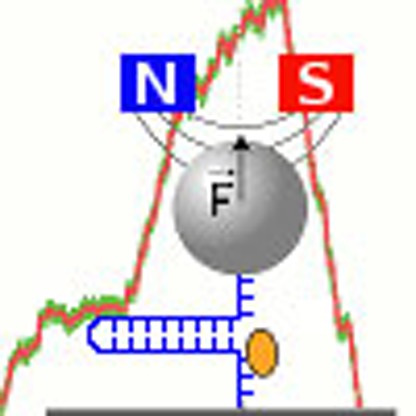

RNA helicases participate in all biological processes involving RNA and are often essential for life^1^. Fuelled by nucleoside triphosphate (NTP), these molecular motors perform mechanical work to unwind double-stranded RNA (dsRNA) or to modulate RNA–protein interactions^2^. Helicases are structurally similar to each other but target a wide range of specific substrates in a precise spatiotemporal manner^3^. However, their exact mechanism of action and the resulting functional impact—as part of large dynamic molecular machinery—remain poorly understood. Upf1 (Up-frameshift 1) is a monomeric RNA helicase belonging to the superfamily 1 (SF1) that is essential for early stages of embryonic development in mice^4^. Upf1 is a multifunctional enzyme involved in different DNA- and RNA-related processes that include DNA repair and replication, telomere homeostasis, histone mRNA turnover and Staufen 1-mediated mRNA decay^5^,^6^,^7^. The best-documented function of Upf1 concerns its role in nonsense-mediated mRNA decay (NMD). NMD is a quality-control mechanism that eliminates mRNAs containing premature translation termination codons (PTC) and also regulates the expression of numerous mRNAs carrying NMD target features^8^,^9^. Upf1 is specifically recruited to NMD targets by an intricate stepwise and translation-dependent pathway^8^. In mammals, several NMD factors including Upf1 and its kinase suppressor with morphogenetic effect on genitalia family member 1 associate with ribosomes stalled on premature translation termination codons and bound to the translation termination factor eRF1-3. In the case of spliced mRNAs, the NMD factors Upf2 and Upf3 are recruited onto mRNAs via the exon junction complexes (EJC) loaded by the splicing machinery^8^,^9^,^10^. When an EJC is located downstream of the stop codon, Upf1 and SMG1 join the EJC with the help of the RNA helicase DHX34 (ref. 11), to form the decay-inducing (DECID) complex in which Upf1 is phosphorylated by SMG1 (ref. 12). Once phosphorylated, Upf1 and the associated NMD factors trigger mRNA decay^9^.

The ATPase activity of Upf1 is required for the correct progression of NMD in all eukaryotic organisms tested so far. Structurally, Upf1 is composed of a characteristic double RecA-like helicase domain (HD) flanked by two external and regulatory domains^13^,^14^. Both domains are inhibitory and maintain the enzyme inactive through distinct mechanisms^14^,^15^. The N-terminal domain, also called CH (rich in Cysteine and Histidine) domain, interacts with the helicase domain to stably wrap the enzyme around the RNA, drastically reducing its helicase activity^13^. The inhibition by the CH domain is relieved by its binding to Upf2 which triggers a large conformational change displacing the CH domain from its blocking position onto the helicase domain^13^. At the C-terminal extremity, the SQ (rich in Serine and Glutamine) domain that carries important phosphorylation sites of Upf1 also makes intramolecular contacts with the helicase domain to impede ATP hydrolysis^14^. In contrast to the CH domain, the mechanism preventing the inhibition by the SQ domain is not yet known. The double inhibition of Upf1 by its external domains is most likely required to keep the enzyme silent until it is activated at the appropriate time frame during NMD. The ATP-dependent activity of Upf1 is notably necessary for complete nonsense mRNA degradation and for the recycling of NMD factors^16^. However, the exact mechanism of action of Upf1 as a potential molecular motor on the targeted messenger ribonucleoprotein (mRNP) remains unknown. All the eukaryotic RNA helicases described so far have been shown to locally unwind nucleic acid structures and to translocate over short distances^17^. In the case of Upf1, it is not known whether it is able to rearrange mRNP nor whether it acts locally or whether it travels along the mRNA.

Here, to investigate the properties of Upf1, we study its behaviour on single RNA and DNA molecules by combining biochemical and biophysical experimental strategies. We find that the helicase domain of Upf1 is capable of both unwinding double-stranded nucleic acids and translocation on single-stranded nucleic acids over long distances. In addition, we compared the activity of Upf1 in the absence and in the presence of its partner Upf2. Finally, we investigated whether the translocation of Upf1 is hampered by the presence of proteins bound to DNA and RNA, revealing an unexpected potential of Upf1.

## Results

### Upf1 unwinds long double-stranded RNA

**Figure 1:**
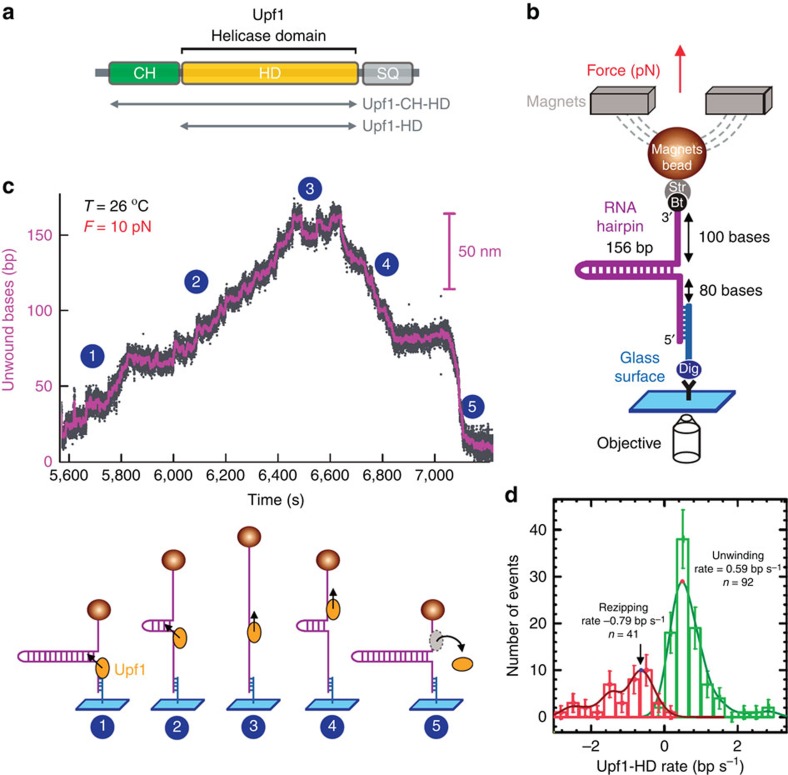
Upf1 helicase activity on RNA. (**a**) Organization of Upf1 and truncated versions (grey arrows) used in this study, Upf1-HD and Upf1. (**b**) Schematic representation of the RNA used for the magnetic tweezers set-up. (**c**) Experimental trace showing the activity of Upf1-HD in saturating concentration of ATP. The number of unwound bases is deduced from the molecular extension *Z*(*t*) obtained at *F*=10 pN. From 5,600 to 6,440 s (steps 1–2) the helicase unwound the 156 bp RNA hairpin. From 6,640 to 7,200 s (steps 4–5) the RNA hairpin refolded, while Upf1-HD translocated on ssRNA reaching the 3′ extremity. (**d**) Distribution of unwinding and translocation rates of Upf1-HD on RNA. (The data corresponds to Upf1 activity observed over 14,400 s, travelling along 2,850 bps in 12 bursts, error bars are s.d.).

We used magnetic tweezers to investigate the hidden mechanical attributes of single molecules of recombinant human Upf1 (Fig. 1a)—a strategy for the first time employed to characterize a eukaryotic RNA helicase. The experimental configuration consisted of an RNA hairpin molecule tethered to a glass surface at its 5′ extremity and to a super-paramagnetic bead through a biotin–streptavidin interaction at its 3′ extremity (Fig. 1b). These molecules are tethered on a glass coverslip, in a thin microfluidic chamber constructed from a slit in a 50 μm double-sided tape joining the glass coverslip and a 50 μm thick Mylar sheet on top. Inlet and outlet ports allow changing the buffer conditions by gentle flows. A pair of magnets was used to apply a constant tension *F* to the hairpin. Adjusting the distance between the magnets and the beads from 2 to 0.125 mm, results in forces ranging between 0.1 and 25 pN respectively. The extension of the molecule with time *Z*(*t*) was obtained by tracking the position of the bead using video-microscopy, which provides an accuracy of ∼3 nm (3–4 bases) between two video frames sampled at 30 Hz. For forces lower than ∼17 pN, the RNA hairpin of 156 bp is fully folded while at higher forces, the hairpin is mechanically unfolded (Supplementary Fig. 1). Under a constant tension, the change in the extension following the opening of one base pair offers a sensitivity of ∼1 nm per bp^18^.

In the presence of Upf1 helicase domain (Upf1-HD) in saturating ATP conditions, we observed that *Z*(*t*) displayed long and characteristic saw-tooth events (Fig. 1c). The burst rising edge corresponds to the unwinding of the hairpin by Upf1-HD (Fig. 1c, position 2). When the helicase reached the hairpin apex (Fig. 1c, position 3), it translocated on the single-stranded RNA (ssRNA; position 4), and the refolding of hairpin was slowed down by the helicase until it ultimately completely refolded (position 5). The Upf1-HD unwinding and translocation phases of different helicase molecules were irregular and presented many pauses. Both phases may be described as series of unwinding (translocation) events interrupted by pauses as shown in Supplementary Fig. 2. The duration of these pauses had a nearly exponential temporal distribution characterized by *τ*_pu_ (*τ*_pt_) (unwinding denoted by u and translocation by t, while p stands for pauses); the duration of the unwinding (translocation) events was also exponentially distributed (*τ*_u_ and *τ*_t_). The values of these characteristic times reported in Supplementary Table 1 were typically in the 10 s range. The unwinding and translocation rates present a broad distribution with a peak typically around 0.59 and −0.79 bp s^−1^, respectively, at 26 °C (Fig. 1d). Upf1-HD translocated faster than it unwound (compare rising and falling edges of events, Fig. 1c). To ensure single-molecule activity, we use low Upf1 concentrations that lead to scarce events which have surprisingly long durations: once a Upf1-HD molecule started unwinding a hairpin it proceeded all the way through the 156 bp substrate for >1,600 s, which are characteristics of a slow but highly processive enzyme (Fig. 1c).

Together with the low enzyme concentration, several pieces of evidence support that a monomer of Upf1-HD unwinds the complete RNA hairpin. First of all, Upf1 has been previously shown to exist as monomer in solution^19^ and we observed that under our experimental conditions, calmodulin-binding peptide-Upf1-HD does not co-precipitate Upf1-HD (Supplementary Fig. 3). In addition, we performed unwinding assays to demonstrate that molecules of Upf1-HD do not work cooperatively. To do so, we constructed three RNA–DNA duplexes of 21 bp preceded by 5′-ssRNA of 5, 12 and 54 nts, respectively (Supplementary Fig. 4a). Unwinding efficiencies were deduced from the proportions of radiolabelled single-stranded DNA (ssDNA) accumulated over time (Supplementary Fig. 4a,b). Upf1 showed a weak unwinding activity with the substrate containing the shortest 5′-overhang that is not long enough to accommodate the binding of one Upf1 molecule that covers around 10 nts^13^. Interestingly, the substrates containing a 5′-overhang long enough to bind one (12 nts) or multiple (54 nts) Upf1 molecules, were unwound with similar efficiencies (Supplementary Fig. 4a,b).

Together, the results from this single-molecule approach showed that the helicase domain of Upf1 is able to both unwind a long dsRNA of 156 bp and translocate onto ssRNA.

### Upf1 unwinds and translocates over long distances

**Figure 2:**
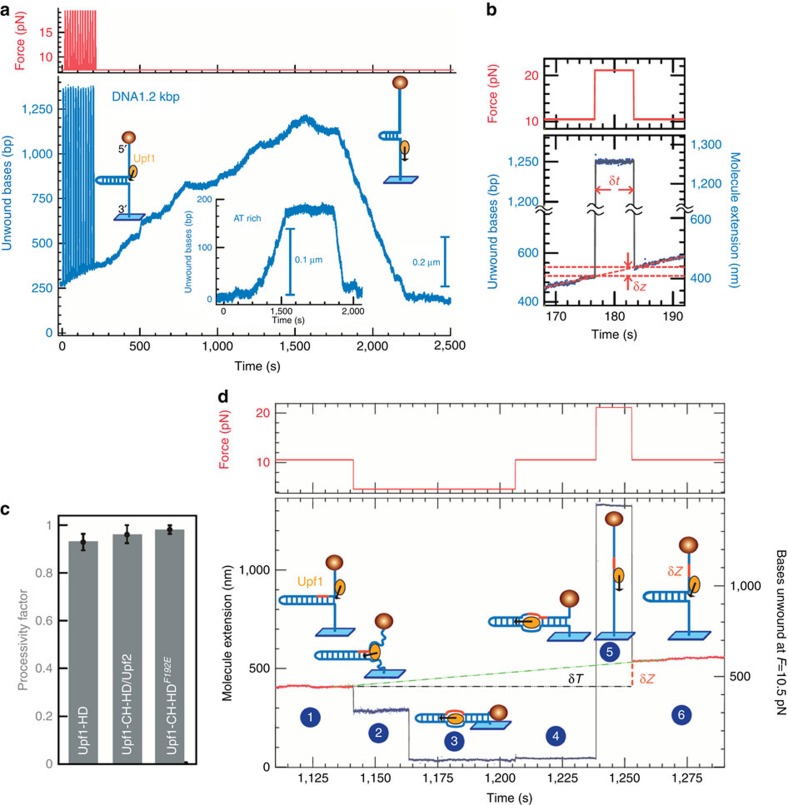
Single-molecule characterization of Upf1 processivity. (**a**) Trace obtained with a 1.2 kb DNA hairpin. A series of force surges (from 0 to 250 s) were applied to the hairpin, exposing single strand, favouring Upf1 binding. This saw-tooth event consists of unwinding (250 and 1,600 s) and ssDNA translocation (1,600 and 2,230 s) of Upf1-HD. (Inset) Similar event with a 180-bps AT-rich DNA hairpin. The GC-rich sequence close to the apex leads to pauses (1,530 and 1,800 s). (**b**) Single strand translocation process observed during a force-jump assay. The amount δ*z* that Upf1-HD has travelled during the force surge lasting time interval δ*t* is read when the hairpin refold. (**c**) Relative processivity of fully active Upf1 forms represented by the processivity factor: Upf1-HD *f*_p_=52/56, Upf1-CH-HD/Upf2 *f*_p_=24/25, Upf1^F192E^ *f*_p_=55/56, error bars are s.d. (**d**) Force modulation (red) in time and molecule extension (blue) showing that Upf1 is active in a DNA bubble. In phases 1 and 5 Upf1-HD unwinds at 10 pN. In phases 2 and 3, *F* was reduced to 5 pN (1,140 and 1,240 s). During a transient (1,140 and 1,164 s) the helicase still blocked the fork, and in phase 3 the unwound ssDNA arms passed over the helicase so that the hairpin fully refolds, enclosing Upf1-HD within a bubble. In phase 4, a force surge to 21 pN (1,240 and 1,252 s) fully re-opens the hairpin. In phase 5, reducing *F* to 10 pN refolds the hairpin partially since the fork is blocked again by Upf1-HD. During the entire time interval (δ*T*) the helicase had slowly progressed within the DNA bubble and was not expelled.

The difficulty in modulating the conditions under which the RNA hairpin is manipulated, combined with Upf1's ability to unwind both dsRNA and double-stranded DNA (dsDNA), as shown by *in vitro* unwinding assays (Supplementary Fig. 4c,d), compelled us to employ much longer DNA hairpins to dissect the activities of Upf1. We used a DNA substrate previously described^20^ that contains a hairpin of 1.2 kbp with a 4-nt loop flanked by a 76 nt 5′-biotinylated ssDNA tail and a 146 bp 3′-digoxigenin-labelled dsDNA tail (Fig. 2a). As for the RNA substrate, we observed with this long DNA hairpin that Upf1-HD could fully unwind the hairpin, pass the apex before translocating onto ssDNA (rezipping phase), until the bead reaches its original position (Fig. 2a). The ability of Upf1 to translocate on ssNA could be directly visualized in a force-jump experiment^21^,^22^. To do so, we applied a force surge of ∼20 pN lasting a duration δ*t* to transiently unzip the hairpin entirely (Fig. 2b). Upf1-HD remained bound to the ssNA and pursued its translocation, as revealed by a change of extension (δ*z*) measured when the force resumed its original value and the hairpin partially refolded (Fig. 2b).

Next, we varied the force applied to the hairpin to determine whether its unwinding and translocation rates were force dependent. Typically, increasing the applied force enables a passive helicase, which waits for a spontaneous thermal opening of the base pairs, to unwind or translocate with higher rates^23^. Interestingly, in the 7–12 pN force range and within our measuring accuracy, Upf1-HD mean rates did not vary (Supplementary Fig. 5a), demonstrating an active mechanism of unwinding^23^ (see Discussion).

As observed on RNA substrate, Upf1-HD also translocated faster than it unwound on DNA. The instantaneous rates of Upf1-HD were irregular (Supplementary Fig. 5b, upper panel), the same alternating pattern of unwinding events interrupted by pauses was observed similar to those observed with the RNA substrate (Supplementary Table 1). The instantaneous unwinding and translocation rates measured without considering the pauses provided slightly higher values (Supplementary Fig. 5b, lower panel and Supplementary Table 1). Pauses during unwinding being longer than the ones during rezipping, explain the mean rate difference between the two phases. While the pauses were more frequent or longer on GC-rich sequences (data not shown), we used an 87% AT-rich hairpin (Fig. 2a, internal panel) to study the rate versus ATP concentration. The unwinding speed of Upf1-HD on this hairpin rises with increasing ATP concentration until it reaches the rate constant *K*_M_ of 1.22±0.2 μM with a saturating speed *V*_max_ of 1.36±0.1 bp s^−1^, showing that the enzyme works at its maximum rate in physiological ATP concentration (Supplementary Fig. 5c).

### Upf1 is a highly processive enzyme

Upf1-HD is a slow translocase compared with other monomeric helicases like UvrD and NS3 when analysed in comparable conditions^24^,^25^. However, it shows a processivity so high that it exceeds the size of the tested hairpins. Upf1-HD reached the end of the 1.2 kb DNA hairpin in 52 unwinding bursts out of 56, and only four bursts were aborted. We calculated a processivity factor *f*_p_ as the ratio of the number of enzymes reaching the hairpin apex over the total number of bursts measured and starting in the first 120 bps of the hairpin (Fig. 2c). Assuming that helicase has a constant probability of detaching in time and sequence, we extrapolated an average processivity of 16±6 kb (see Methods). This property indicates a very strong interaction between Upf1-HD and ssDNA, as further confirmed by a low-force experiment (Fig. 2d). During the course of an unwinding event initiated at *F*=10 pN (Fig. 2d, position 1), the force was reduced to 5 pN that induced after a lag time (Fig. 2d, position 2), the complete refolding of the hairpin (Fig. 2d, position 3). This delay in refolding, corresponds to the time needed before the occurrence of a thermal fluctuation strong enough to bring the two arms of the fork to encircle the helicase (at 5 pN the time distribution of such an event is exponential as seen in Supplementary Fig. 5d). Surprisingly, when the hairpin was refolded at 5 pN (Fig. 2d, positions 4 and 5), Upf1-HD was not ejected from the DNA but rather entrapped within a DNA bubble. Indeed, after a few minutes, we applied a force surge of 20 pN to open the hairpin (Fig. 2d, position 5), followed by a phase at 10 pN refolding it (Fig. 2d, positions 6). Under these conditions, the blocking position revealed the new Upf1-HD location to be shifted forward by an amount δ*z*, corresponding to the enzyme progression during time interval δ*t*. Repeating this process many times demonstrated that the helicase remained bound to one strand and pursued translocation inside a NA bubble when the hairpin was closed Supplementary Fig. 6. These data show the very tight association of the enzyme with NA explaining its high processivity.

### Upf2-mediated activation at single-molecule level

**Figure 3:**
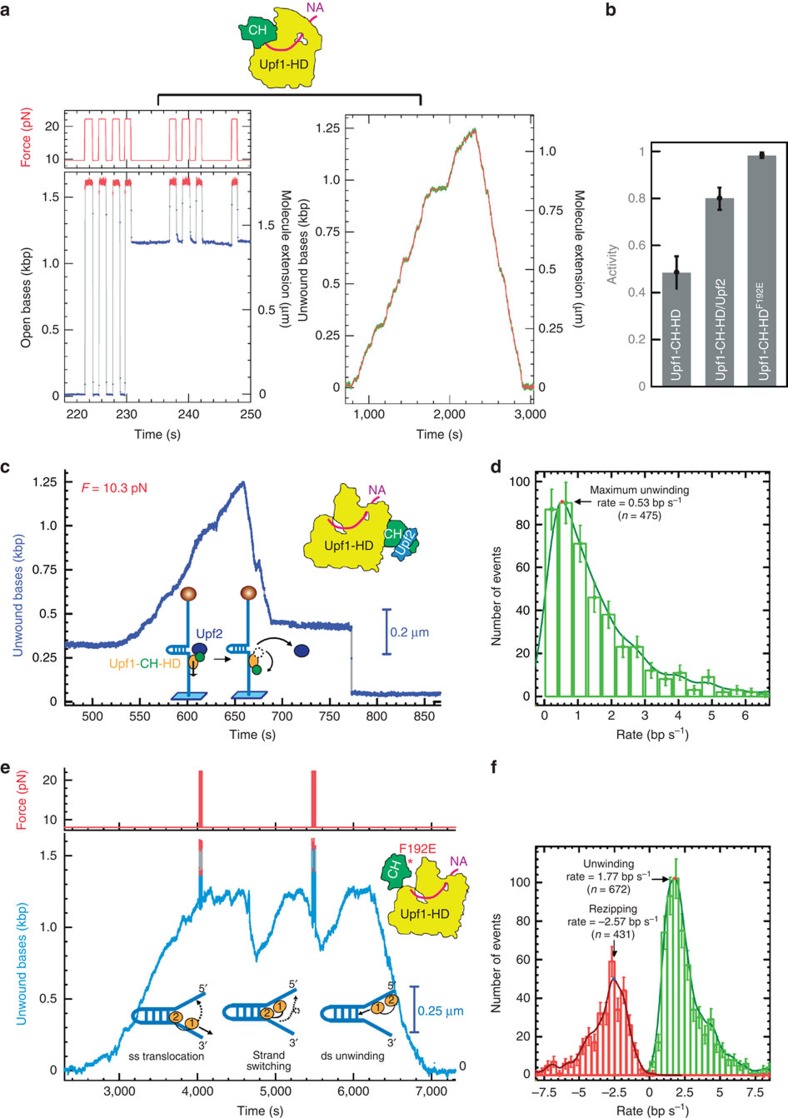
The binding of Upf2 to CH domain activates Upf1-CH-HD unwinding and translocation. (**a**) Experimental magnetic tweezers traces corresponding to the two types of enzymatic activity detected for Upf1 on DNA hairpin. On the left, Upf1 binds on ssDNA (starting at 231 s), blocking the rezipping of the hairpin. On the right the enzyme is active. (**b**) Histogram of relative activity of Upf1-CH-HD, Upf1-CH-HD/Upf2 complex and Upf1^F192E^ mutant (see text, error bars are s.d.). (**c**) Trace of human Upf1-CH-HD/Upf2 complex (left panel) unwinding steadily and completely the DNA molecule, passing the apex, pursuing its translocation and refolding the DNA hairpin. At *t*=675 s, the complex makes two strand-switching events before finally stopping its activity leaving the hairpin blocked (at *t*=687 s). This trace is atypical since the Upf1-CH-HD/Upf2 complex translocates for some time before stalling; for most bursts, the complex stops very quickly after passing the apex. (**d**) Distribution of instantaneous unwinding rate of the Upf1-CH-HD/Upf2 complex; the number of translocation events is too small to allow the same measurement (error bars are s.d.) (**e**) Experimental magnetic tweezers trace showing the molecular extension as a function of time obtained for the Upf1^F192E^ mutant on a DNA hairpin. Notably, during translocation on ssDNA (*t*=4,700 and 5,500 s) Upf1^F192E^ switches strands before the hairpin is completely refolded and starts a new unwinding run followed by a translocation. (**f**) Distribution of unwinding and translocation velocities of Upf1^F192E^ (error bars are s.d).

We next analysed at the single-molecule level, a larger version of Upf1 containing both its CH regulatory domain and its helicase domain, referred to as Upf1-CH-HD (Fig. 1a). The CH domain allosterically increases the affinity of the protein for the NA, reducing its helicase activity^13^. We observed two different behaviours of Upf1-CH-HD molecules (Fig. 3a). Some molecules completely blocked the hairpin refolding after their random binding onto ssDNA (Fig. 3a, left panel). The blocking position remained constant, showing that the enzyme binds but does not translocate. In other cases, we observed an activity of Upf1-CH-HD (Fig. 3a, right panel) comparable to the activity of Upf1-HD. Such activity may be attributed to molecules in which the inhibitory CH domain is flipped out from HD. Analysis of multiple events revealed that about 50% of Upf1-CH-HD remained bound to DNA but immobile, without unwinding activity (Fig. 3b) while the other 50% travelled like Upf1-HD, in agreement with the twofold reduction of the activity of Upf1-CH-HD compared with Upf1-HD in solution^13^. As expected, when Upf2 was added, or when the mutant of Upf1^F192E^ preventing the inhibitory effect the CH domain^12^ was tested, almost all Upf1 molecules became as active as Upf1-HD (Fig. 3a,c,d). Following a strict protocol (see Methods), we have characterized activity versus blocking by the ratio Ac*=u*/(*u+b*) where *u* equals the number of unwinding events and *b* equals the number of binding events. For Upf1-CH-HD, Ac=49% (see Supplementary Table 2 for details). For Upf1-CH-HD/Upf2, Ac=81%. This was confirmed by analysing in the same condition the mutant Upf1^F192E^. For Upf1^F192E^, Ac=99.1%.

For 76±8% of the Upf1-CH-HD/Upf2 binding events, we noticed a characteristic burst shape with a normal rising edge but a falling edge interrupted by blocking (Fig. 3c at 770 s). So, Upf1-CH-HD/Upf2 complex unwound the hairpin completely, and started translocating on ssDNA before stopping after about 100 s. This event suggests that once the complex passed the apex, the fork pressure quickly ejected Upf2 from Upf1 (Fig. 3c). As a result, the unwinding rate of Upf1-CH-HD/Upf2 can be measured (Fig. 3d), but the statistics on translocation is too low to provide a reasonable rate. This scenario was confirmed by assaying the mutant Upf1^F192E^, with which no blocking of the enzyme has been observed (Fig. 3e). Upf1^F192E^ appears to have more regular rates than Upf1-HD or Upf1-CH-HD/Upf2 complex, presenting fewer pauses (Supplementary Table 2). The mean rates of both unwinding and translocation are somewhat higher as seen in Fig. 3f. Interestingly, this mutant revealed another feature of Upf1^F192E^. Often, this mutant unwound the hairpin (as Upf1-CH-HD/Upf2 complex), passed the apex and started translocating but it rapidly performed a strand-switching event. In this process the helicase direction was reversed and the helicase started unwinding the hairpin again (Fig. 3e at 4,800 and 5,600 s). This strand-switching mechanism severely reduced the processivity of Upf1^F192E^ in the rewinding phase. As seen in Supplementary Fig. 7, the processivity is only 290 bp in the translocation mode but it is even larger than the processivity of Upf1-HD in the unwinding phase as seen in Fig. 2c. As a result Upf1^F192E^ definitely prefers to unwind a dsDNA than to translocate. This strand switching was also observed for Upf1-HD and Upf1-CH-HD/Upf2 but less frequently (data not shown).

Therefore, our single-molecule approach confirms that Upf2 activates Upf1-CH-HD, and reveals that the enzyme bound to its activator is processive, with a highly efficient activation mechanism (95%).

### Upf1 remodels nucleoprotein complexes

**Figure 4:**
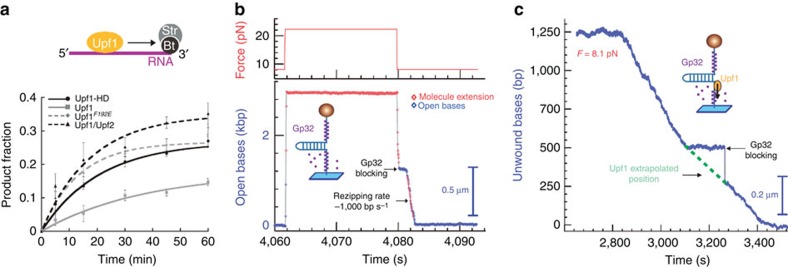
Active translocating Upf1 disrupts protein–NA interactions. (**a**) Time course for streptavidin displacement from 3′-biotinylated RNA by Upf1-HD translocation. Data points (Supplementary Fig. S5a) were fitted with *y*=*A*(1−*e*^*–kt*^)^57^ (error bars are s.d.) (**b**) Force-jump experiment showing that Gp32-B (300 nM) covers ssDNA. In the absence of Upf1, when the hairpin is open, Gp32-B binds DNA, as seen by the slowdown of the DNA hairpin refolding (from 4,080 s to about 4,082 s, *F*=8 pN, refolding rate of 1,000 bp s^−1^). Gp32-B is known to efficiently bind ssDNA with a dissociation rate measured as ∼1 molecule per second on 65 bp s^−1^ (refs 58, 59). (**c**) Single-molecule analysis of Gp32-B displacement from ssDNA by translocating Upf1-HD (from 2,840 to 3,480 s) The Gp32-B protein was stripped off from ssDNA in presence of Upf1-HD and ATP (right panel). During Upf1 translocation, Gp32-B attached on the displaced strand may transiently block the fork refolding as seen in the time window (from 3,080 to 3,270 s).

Finally, to investigate the possible nucleoprotein remodelling activity of Upf1, we tested whether the translocation of Upf1 was hampered by the presence of proteins on the substrate. We first performed a streptavidin displacement assay in which we monitored the ability of Upf1-HD to displace a streptavidin molecule bound to a biotinylated RNA^26^. Briefly, the enzyme and the substrate were first pre-incubated before initiation of the reaction by adding ATP, magnesium and an excess of biotin to trap free streptavidin molecules. Time-course aliquots were analysed on native polyacrylamide gel to separate free RNA from streptavidin-bound RNAs. Remarkably, in presence of ATP only, Upf1-HD, Upf1-CH-HD/Upf2 complex and Upf1^F192E^ efficiently displaced the streptavidin with a comparable efficiency while Upf1-CH-HD showed a much weaker activity (Fig. 4a and Supplementary Fig. 8a,b).

To confirm this Upf1 property, we analysed the progression of Upf1-HD on single molecules of DNA covered by the ssDNA-binding protein Gp32 from the T4 phage. More precisely, we employed the mutant Gp32-B that avidly binds ssDNA without cooperative binding^27^. At high force (22 pN) the DNA hairpin was fully opened and incubated with a large excess of Gp32-B to allow its binding (Fig. 4b). The binding of Gp32-B on the opened DNA hairpin is easily detected when refolding the hairpin by lowering the force: the refolding is clearly slower and less regular than in the absence of Gp32-B, since the fork needs to eject bound Gp32-B (Fig. 4b). This assay has already been done over a wider concentration range demonstrating that Gp32 coats ssDNA and the fork removes it as the hairpin refolds^28^. A similar behaviour was also observed for ribosomal protein S1 (ref. 29). The slowing down of the refolding increases with Gp32 concentration. Interestingly, the unwinding and translocation activities of Upf1-HD were not altered by the presence of Gp32-B (Fig. 4c). These data demonstrate that the slow but processive translocase activity of Upf1 is sufficient to remodel NA–protein interactions. The pause in the refolding at *t*=3,100 s demonstrates that Gp32-B on the opposite strand has transiently prevented the hairpin refolding; meanwhile the helicase keeps translocating along the ssDNA at the normal rate until the refolding fork reaches again the motor at *t*=3,270 s (Fig. 4c). We could not detect extra pausing of Upf1 induced by Gp32-B.

## Discussion

Helicases can carry different biophysical properties including RNA binding, translocation onto ssNA or dsNA, dsNA separation and RNA–protein remodelling over more or less long distance ranging from few nucleotides to several kilobases^30^,^31^. Therefore, the term ‘helicase' encompasses a large variety of molecular motors, and a thorough understanding of their mechanism of activity necessitates the development of different and complementary experimental strategies. Our single-molecule approach using magnetic tweezers combined with biochemical assays constitute powerful tools for revealing the multiple facets of Upf1, and notably its highly processive translocation propensity. So far, the only eukaryotic RNA helicases known to translocate was the human SF2 RIG-I RNA helicase involved in antiviral immune response, which is able to translocate onto dsRNA without unwinding the duplex^32^. Here, we bring to light a novel and different example by showing that human Upf1 translocates onto ssNA and hence, it is capable of both melting long dsNA and remodelling stable NA–protein interactions. The comparison of Upf1 with other helicases revealed the following peculiar properties of Upf1. (1) Upf1 is phylogenetically close to the SF1 DNA helicase UvrD, playing a key role in DNA repair and plasmid replication in bacteria. In single-molecule experiments, UvrD unwinds DNA with a velocity in the range of 10 nts s^−1^ (refs 24). Upf1 also shares mechanical features with the hepatitis C virus SF2 RNA helicase NS3 that translocates with a velocity in the range of 50 nts s^−1^ (excluding pauses)^34^,^35^. Therefore, Upf1 is a slow helicase that progresses at least an order of magnitude slower that UvrD and NS3 studied with similar approaches. (2) Helicases can be considered as passive or active enzymes^23^,^36^,^37^. In fact, in the active mechanism the helicase contains a sub-domain that actively destabilizes the duplex while the helicase motor pushes the enzyme as previously observed for PcrA^38^. In that case, the melted duplex opens faster than the melting caused by normal thermal fluctuations, so the enzyme rate is limited mainly by the motor biochemistry and is constant, independent of the force applied to the NA fork. We consider that Upf1 is an active helicase because its unwinding rate is independent of the force applied to the substrate extremities (Supplementary Fig. 4a). (3) A common property of Upf1, NS3 and UvrD is their ability to switch between close templates^24^,^25^. While the biological significance of strand switching is still unclear, its occurrence could be explained by an inchworm mechanism of translocation^38^. This model postulates the existence of two nucleic acid-binding sites, each located on a RecA-like domain (RecA1 and RecA2 in Upf1). These two domains switch alternatively between tight and weak NA-binding states and move relative to each other in response to ATP hydrolysis cycles^38^. During the unwinding phase, while Upf1 is translocating onto one strand, one of its RecA-like domains may contact the opposite strand followed by the second RecA-like domain, leading to unwinding reinitiation on the 5′ side of the hairpin. (4) Our study also revealed the remarkable processivity of Upf1, which exceeds several kilobase pairs without detaching from its substrate. Interestingly, the processivity of Upf1 is comparable to the one of *Escherichia coli* RecBCD^39^ despite the fact that these two motors must work differently. To maintain its high processivity, RecBCD uses two motor units, RecB and RecD. Each motor progresses on opposite strands with opposite polarity and both form a tunnel around DNA ensuring a tight binding^40^. Working as a monomer, the processivity of Upf1 can neither be explained by a comparable bipolar translocation, nor by encircling one strand. Its processivity most likely results from the exceptional binding affinity of both RecA domains with NA. The tight binding of Upf1 with the strand on which it progresses is demonstrated by the ability of Upf1 to pursue translocation after the refolding of the hairpin (Fig. 2d and Supplementary Fig. 6), a unique feature not seen with any other helicase so far. On the other hand this strong binding may explain the slow rate of this helicase. The precise role played by Upf1 during DNA replication^5^,^6^ remains unknown but its ability to travel onto DNA over long distance is most likely important for its function. (5) Our approaches allowed determining the ability of Upf1 to overcome obstacles encountered during translocation. The outcome of the encounter between a translocase and ssDNA-binding proteins was analysed for the SF2 helicase XPD^41^. Unlike Xeroderma pigmentosum group D helicase, the Upf1 translocation rate was not slowed down by the presence of Gp32-B on the ssDNA. Moreover, we showed that Upf1 could displace a streptavidin block from the RNA substrate. We suppose that the tight association of Upf1 with NA while it is translocating provides a force sufficient to strip-off a wide range of RNA binding proteins bound to RNA. This property is certainly important for its role in the RNA related mechanism to which it participates and notably NMD.

**Figure 5:**
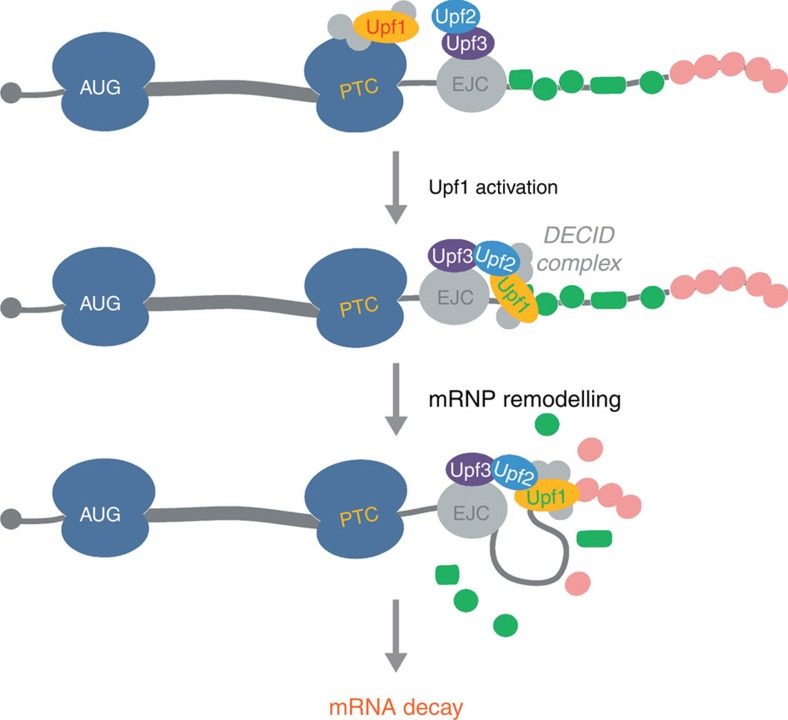
Model proposed for the late phases of NMD. On premature translation termination, Upf1 and associated factors are recruited by stall ribosomes. At this stage, the helicase Upf1 (written in red) is inactive. Then, Upf1 joins Upf2 and Upf3 bound to an EJC to form the DECID complex is which Upf1 (written in green) is activated. Finally, being highly processive, Upf1 translocates toward the 3′-end extremity of the mRNA to remodel the mRNP leading to mRNA decay.

In eukaryotes, >70 RNA helicases exist, which are involved in almost all RNA-dependent processes^17^. Large machineries like the spliceosome and the ribosome require several helicases for their biogenesis and their function^42^,^43^. Understanding both the action and the regulation of helicases constitutes an important step to dissect how these machineries work. At least three helicases participate in NMD: DHX34, the DEAD-box helicase eIF4A3 (DDX48) as the central component of the EJC core and Upf1 (refs 11, 44, 45). In contrast to the two first helicases, Upf1 is essential for NMD. However, several questions remain open—notably concerning Upf1 recruitment to NMD targets, the timing of its binding to RNA and its activation and finally its mode of action as a helicase. Recent studies aiming at identifying the RNA targets of Upf1 in mammals, preferentially detected Upf1 along the entire 3′ untranslated region (3′-UTR) of NMD targets and not in the vicinity of the termination codon^46^,^47^,^48^,^49^,^50^,^51^. This picture of Upf1 binding sites may reflect the random binding of Upf1 over the 3′-UTR that is more accessible than the coding region from which RNA binding proteins get displaced by translating ribosomes. Alternatively, it may correspond to snapshot of Upf1 while it is translocating along the 3′-UTR. The fact that Upf1 when repressed by its flanking domains forms a stable clamp, while the activated form slowly translocates over very long distances (Fig. 3), would argue for the second scenario. Also in agreement with this scenario, isolated Upf1 binding sites are enriched upstream of sequences with a higher propensity to form secondary structure and G-rich regions^48^. Interestingly, our *in vitro* approach detected identical trends since single molecules of Upf1 unwind dsRNA molecules more slowly than they translocate (Fig. 1) and make pauses more frequently on GC-rich substrates (Fig. 2). Upf1 operates as molecular motor most likely during the late phases of NMD. Briefly, after its recruitment by prematurely terminating ribosomes, Upf1 joins the downstream EJC bound to Upf3 and Upf2 to constitute the DECID complex in which Upf1 is phosphorylated^8^,^9^,^12^. Concomitantly, although Upf1 activity is repressed by its two regulatory domains, it is enzymatically turned on notably by its binding to Upf2 and its phosphorylation^13^,^14^,^49^. In this complex, Upf1 binds RNA towards the 3′-end^52^. When the targeted mRNA is cleaved by the endonuclease SMG6, the helicase activity of Upf1 is required for the disassembly of the 3′ portion of the mRNP and the completion of decay^16^. The remodelling activity of Upf1, combined with its remarkable processivity may also serve to rearrange the mRNP far downstream the stop codon paving the way for RNA degradation, as illustrated in Fig. 5. Each mRNA is packed in a specific particle made of a complex set of RBPs essential for finely tune mRNA localization, translation and decay^53^. Long-range remodelling by Upf1 may irreversibly affect the fragile equilibrium of mRNP^54^ and push the mRNA towards degradation.

While Upf1 is the first eukaryotic monomeric RNA helicase proved to be highly processive, other helicases probably possess similar properties. One obvious candidate is the closely related SF1 helicase Mov10 also enriched on the 3′-UTR region of multiple transcripts including some NMD targets bound to Upf1 (ref. 46). Whether Upf1 and Mov10 employ their helicase activity to perform distinct or redundant actions remains an open question.

## Methods

### Proteins

Human Upf1-HD (295–914 amino acids), Upf1-CH-HD (115–914 amino acids) Upf1-CH-HD-F192E and Upf2 (761–1227 amino acids; Fig. 1a) proteins were cloned in a pet28a (Novagen) derivative plasmid and expressed in *E.coli* BL21(DE3) Rosetta cells. The Upf1 protein truncations were CBP-tagged at the N-terminal and His-tagged at the C-terminal, while Upf2 was His tagged. The cell cultures were grown in 1 l of LB medium and induced overnight at 16 °C. The cells were lysed in buffer A (1.5 × PBS pH 7.5, 225 mM NaCl, 1 mM magnesium acetate, 0.1%(w/v) NP-40, 20 mM imidazole and 10%(w/v) glycerol) supplemented with 100 μg ml^−1^ of egg white lysozyme (Sigma-Aldrich) and with 1 × protease inhibitor cocktail EDTA-Free (Sigma-Aldrich). Soluble lysate was applied to a prepacked nickel column (HisTrap FF crude, GE Healthcare) and fractioned on an Äkta Purifier (GE Healthcare) using a linear gradient from buffer A to B (buffer A added of 0.5 M imidazole) over 20 column volumes. The CBP-fused proteins were subsequently purified on a Calmodulin affinity column. Loading of the samples and column washes were performed with a buffer C (1 × PBS pH 7.5, 150 mM NaCl, 1 mM magnesium acetate, 0.1%(w/v) NP-40, 1 mM DTT, 4 mM calcium chloride and 10%(w/v) glycerol) and elution with the same buffer containing 20 mM EGTA instead of calcium chloride^13^,^14^,^15^. To assemble the Upf1-CH-HD/Upf2 complex, 50 nM of both proteins were incubated for 2 h at 4 °C in helicase buffer (20 mM Tris-HCl pH 7.5, 50 mM potassium acetate, 2 mM MgCl_2_, 2% BSA, 1 mM DTT, 0,1 mM EDTA). The Gp32-B protein was cloned in petIMPACT plasmid and expressed into *E.coli* BL21(DE3) cells. The protein purification was performed using a chitin column and analysed for purity using SDS–polyacrylamide gel electrophoresis^55^.

### RNA hairpin production

A shorter palindromic sequence was produced from pLacD1 plasmid^34^ and used as a substrate for RNA synthesis. Two DNA fragments were amplified from pLacD1 by PCR using two pairs of primers HLH1853/HLH2124 and HLH2124/HLH2125 to produce DNA1 and DNA2, respectively (Supplementary Table 3 and Supplementary Fig. 9a). DNA1 included the first half of the hairpin (156 bp) followed by 105 bp of extra DNA region and an upstream T7 promoter sequence. DNA2 included the second half of the hairpin and 100 extra bp. Both fragments were gel purified and sequenced separately. DNA1 and DNA2 were digested with BglII and ligated together (T4 DNA ligase, New England Biolabs). The resulting fragment was digested with EcoRI and HindIII and cloned into the corresponding sites of pBR322 to originate pHL1030 plasmid (T4 DNA ligase, New England Biolabs; Supplementary Fig. 9a). A transcription template was obtained by digestion of 1 μg of pHL1030 with HindIII followed by phenol–chloroform extraction and ethanol precipitation (Supplementary Fig. 9b). The DNA template was *in vitro* transcribed using the HLH1164 oligonucleotide (MegaScript T7 kit, Life Technologies) and the RNA product was purified on denaturing (8 M urea) polyacrylamide (5%) gel as previously described^14^,^15^. To introduce biotin moieties at 3′ terminal end of the hairpins, the RNA pellet (60 μg) was re-suspended in 42 μl of diethylpyrocarbonate-treated water before addition of 80 μg of sodium periodate and incubated 90 min at 4 °C in the dark before adding 0.4 μg of biotinamidocaproyl hydrazide (Sigma-Aldrich) followed by an incubation for 20 min at 22 °C in the dark. Then, 0.25 mg of sodium cyanoborohydride was added before incubation for 90 min at 22 °C. The sample was purified twice on G50 column (Bio-Rad) before phenol–chloroform extraction and ethanol precipitation. The pellet was washed with 70% ethanol and re-suspended in DEPC-treated water. The 3′-biotinylated RNA was annealed to 37-nt oligonucleotide handle containing 3′ digoxigenin group (HLH2181, Eurogenetec) The mixture containing 1 μM of RNA/DNA hybrid, 20 mM of MES pH 6.5 and 3 mM magnesium chloride, was heated for 4 min at 95 °C then slowly cooled at 18 °C for annealing. The length and the pairing of RNA stem was confirmed as described elsewhere^20^.

### DNA substrates for single-molecule measurements

The DNA 1.2 kbp substrate used in the single-molecule studies is a 1,239 bp hairpin with a 4-nt loop, a 76-nt 5′-biotinylated ssDNA tail and a 146 bp 3′-digoxigenin-labelled dsDNA tail^20^. The shorter DNA hairpin substrates were all synthesized by hybridizing and ligating oligonucleotides (Eurogentec) as depicted in Supplementary Fig. 10. The oligonucleotide sequences are listed in Supplementary Table 3. Briefly, two oligonucleotides (B1 and B2) were annealed to form a stem. A short hairpin (oligonucleotide C) and two ssDNA arms (oligonucleotides A1 and A3) were annealed and ligated (T4 DNA ligase, New England Biolabs) to the compatible ends of the stem. The primer A1 contained biotin at the 5′ end to attach the molecule to magnetic bead and A3 was modified by adding digoxigenin at the 3′ end for the binding to the glass surface. The digoxigenin label was incorporated by annealing the primer (A2) to the template strand and filling in the overhang with Klenow Fragment (3′→5′ exo-, New England Biolabs) in the presence of dUTP-digoxigenin (Roche). The hairpin products were purified with NucleoSpin Extract II Kits (Clontech). In order to synthesize the AT DNA hairpin the oligonucleotides B1 and B2 were replaced by B3 and B4, respectively. The GC-rich hairpin synthesis has been previously described^56^.

### Experiment with magnetic tweezers

We used a PicoTwist magnetic tweezers instrument (www.picotwist.com) to manipulate individual DNA and RNA hairpin molecules. The DNA hairpins were attached by the 5′ biotinylated extremity to streptavidin-coated magnetic beads (Dynabeads MyOne streptavidin T1, Life Technology) and by a 3′ digoxigenin modified extremity to a anti-Dig-coated glass surface. The RNA hairpins were attached to the magnetic bead by the 3′ biotinylated extremity and to a glass surface via a digoxigenin modified oligonucleotide handle. For both DNA and RNA substrates, the glass coverslip was treated with anti-digoxigenin antibody (Roche) and passivated with 1 × P Buffer (1X PBS pH 7.5, 0.2% pluronic surfactant, 5 mM EDTA, 10 mM sodium azyde, 1 U μl^−1^ of RNase inhibitor (Ribolock, Thermo scientific) and 0.2% of BSA (Sigma-Aldrich). The beads were trapped in the magnetic field generated by a pair of magnets located above the reaction chamber. Forces in the pN range were calibrated using the horizontal Brownian motion^21^,^22^.

### Single-molecule helicase assay

Experiments were conducted at 37 °C for DNA and 26 °C for the RNA, unless otherwise mentioned. The helicase buffer was 20 mM Tris-HCl pH 7.5, 75 mM potassium acetate, 3 mM magnesium chloride, 2% BSA, 0.5 mM DTT, and 2 mM ATP. The Upf1 concentration indicated was the lowest possible to observe helicase activity in single-molecule conditions. During the helicase assays, DNA hairpins and RNA hairpins were maintained with a constant force in the range of 8–12 pN.

### Single-molecule data analysis

Raw data, corresponding to the real-time evolution of the DNA extension in nanometre, was converted into number of unwound base pairs using a calibration factor determined by the elastic properties of ssDNA or ssRNA.

### *In vitro* helicase assay

RNA–DNA hybrids were made with the same DNA oligonucleotide of 21 nt (HLH 1172) annealed to three different complementary RNA to form 5′ ssRNA overhang of 5, 12 and 54 nt (Supplementary Fig. 3a). Transcription of the RNAs, radiolabelling of DNA oligonucleotide, purification of the substrates and unwinding assay were performed as previously described^14^. The oligonucleotides used to produce RNA substrates are listed in Supplementary Table 3 (HLH1164, HLH1376, HLH1377 and HLH2653).

### Upf1/Upf2 activity protocol

To measure activity we have used the following protocol: any traces shorter than 1,000 s or those presenting no binding or no unwinding were disregarded. From the remaining bead traces, we selected those where the enzyme was seen unwinding the hairpin (as in Fig. 3a right) from those where the hairpin presents a blockage by a stationary helicase (as in Fig. 3a left). The activity was defined as Ac=*u*/(*u+b*) where *u* equals the number of unwinding events and *b* equals the number of binding events.

### Streptavidin displacement assay

The streptavidin displacement assay was performed using the helicase domain of Upf1 on a 3′-biotinylated 30-mer RNA substarte bound to a Streptavidin monomer (Promega). The mixture is prepared by mixing 1 nM of the substrate with 60 nm of Upf1-HD enzyme in a F-100 buffer (20 mM MES pH 6.0, 100 mM potassium acetate, 1 mM DTT, 0.1 mM EDTA). A preincubation of 5′ was performed before the enzymatic reaction was initiated by adding 2 mM ATP together with 4 μM of free biotin.At defined incubation times, 2 μl of reaction aliquots were mixed with 5 μl of quench buffer (150 mM NaAc, 10 mM EDTA, 0.5% (w/v) SDS, 25% (w/v) Ficoll-400, 0.05% (w/v) xylene cyanole and 0.05% (w/v) bromophenol blue) in a preset tubes, and immediately loaded on the running gel. Samples were separated by 8% (w/v) polyacrylamide gel containing 0.3% of SDS and detected using a Typhoon 9400 phosphorimaging system and the ImageQuant software (GE Healthcare).

## Additional information

**How to cite this article**: Fiorini, F. *et al.* Human Upf1 is a highly processive RNA helicase and translocase with RNP remodelling activities. *Nat. Commun.* 6:7581 doi: 10.1038/ncomms8581 (2015).

## Supplementary Material

Supplementary Information Supplementary Figures 1-10 and Supplementary Tables 1-3

## References

[b1] Fairman-Williams M. E., Guenther U. P. & Jankowsky E. SF1 and SF2 helicases: family matters. Curr. Opin. Struct. Biol. 20, 313–324 (2010). 20456941 10.1016/j.sbi.2010.03.011 PMC2916977

[b2] Lohman T. M., Tomko E. J. & Wu C. G. Non-hexameric DNA helicases and translocases: mechanisms and regulation. Nat. Rev. Mol. Cell. Biol. 9, 391–401 (2008). 18414490 10.1038/nrm2394

[b3] Singleton M. R., Dillingham M. S. & Wigley D. B. Structure and mechanism of helicases and nucleic acid translocases. Annu. Rev. Biochem. 76, 23–50 (2007). 17506634 10.1146/annurev.biochem.76.052305.115300

[b4] Medghalchi S. M. *et al.* Rent1, a trans-effector of nonsense-mediated mRNA decay, is essential for mammalian embryonic viability. Hum. Mol. Genet. 10, 99–105 (2001). 11152657 10.1093/hmg/10.2.99

[b5] Azzalin C. M. & Lingner J. The human RNA surveillance factor UPF1 is required for S phase progression and genome stability. Curr. Biol. 16, 433–439 (2006). 16488880 10.1016/j.cub.2006.01.018

[b6] Chawla R. *et al.* Human UPF1 interacts with TPP1 and telomerase and sustains telomere leading-strand replication. EMBO J. 30, 4047–4058 (2011). 21829167 10.1038/emboj.2011.280 PMC3209776

[b7] Kim Y. K., Furic L., Desgroseillers L. & Maquat L. E. Mammalian Staufen1 recruits Upf1 to specific mRNA 3'UTRs so as to elicit mRNA decay. Cell 120, 195–208 (2005). 15680326 10.1016/j.cell.2004.11.050

[b8] Kervestin S. & Jacobson A. NMD: a multifaceted response to premature translational termination. Nat. Rev. Mol. Cell. Biol. 13, 700–712 (2012). 23072888 10.1038/nrm3454 PMC3970730

[b9] Popp M. W. & Maquat L. E. Organizing principles of mammalian nonsense-mediated mRNA decay. Annu. Rev. Genet. 47, 139–165 (2013). 24274751 10.1146/annurev-genet-111212-133424 PMC4148824

[b10] Schweingruber C., Rufener S. C., Zund D., Yamashita A. & Muhlemann O. Nonsense-mediated mRNA decay—mechanisms of substrate mRNA recognition and degradation in mammalian cells. Biochim. Biophys. Acta 1829, 612–623 (2013). 23435113 10.1016/j.bbagrm.2013.02.005

[b11] Hug N. & Caceres J. F. The RNA helicase DHX34 activates NMD by promoting a transition from the surveillance to the decay-inducing complex. Cell Rep. 8, 1845–1856 (2014). 25220460 10.1016/j.celrep.2014.08.020 PMC4534575

[b12] Kashima I. *et al.* Binding of a novel SMG-1-Upf1-eRF1-eRF3 complex (SURF) to the exon junction complex triggers Upf1 phosphorylation and nonsense-mediated mRNA decay. Genes Dev. 20, 355–367 (2006). 16452507 10.1101/gad.1389006 PMC1361706

[b13] Chakrabarti S. *et al.* Molecular mechanisms for the RNA-dependent ATPase activity of Upf1 and its regulation by Upf2. Mol. Cell. 41, 693–703 (2011). 21419344 10.1016/j.molcel.2011.02.010

[b14] Fiorini F., Boudvillain M. & Le Hir H. Tight intramolecular regulation of the human Upf1 helicase by its N- and C-terminal domains. Nucleic Acids Res. 41, 2404–2415 (2013). 23275559 10.1093/nar/gks1320 PMC3575847

[b15] Chamieh H., Ballut L., Bonneau F. & Le Hir H. NMD factors UPF2 and UPF3 bridge UPF1 to the exon junction complex and stimulate its RNA helicase activity. Nat. Struct. Mol. Biol. 15, 85–93 (2008). 18066079 10.1038/nsmb1330

[b16] Franks T. M., Singh G. & Lykke-Andersen J. Upf1 ATPase-dependent mRNP disassembly is required for completion of nonsense- mediated mRNA decay. Cell 143, 938–950 (2010). 21145460 10.1016/j.cell.2010.11.043 PMC3357093

[b17] Jankowsky E. RNA helicases at work: binding and rearranging. Trends Biochem. Sci. 36, 19–29 (2011). 20813532 10.1016/j.tibs.2010.07.008 PMC3017212

[b18] Essevaz-Roulet B., Bockelmann U. & Heslot F. Mechanical separation of the complementary strands of DNA. Proc. Natl Acad. Sci. USA 94, 11935–11940 (1997). 9342340 10.1073/pnas.94.22.11935 PMC23661

[b19] Bhattacharya A. *et al.* Characterization of the biochemical properties of the human Upf1 gene product that is involved in nonsense-mediated mRNA decay. RNA 6, 1226–1235 (2000). 10999600 10.1017/s1355838200000546 PMC1369996

[b20] Manosas M., Spiering M. M., Zhuang Z., Benkovic S. J. & Croquette V. Coupling DNA unwinding activity with primer synthesis in the bacteriophage T4 primosome. Nat. Chem. Biol. 5, 904–912 (2009). 19838204 10.1038/nchembio.236 PMC2784132

[b21] Lionnet T., Spiering M. M., Benkovic S. J., Bensimon D. & Croquette V. Real-time observation of bacteriophage T4 gp41 helicase reveals an unwinding mechanism. Proc. Natl Acad. Sci. USA 104, 19790–19795 (2007). 18077411 10.1073/pnas.0709793104 PMC2148377

[b22] Manosas M. *et al.* Magnetic tweezers for the study of DNA tracking motors. Methods Enzymol. 475, 297–320 (2010). 20627163 10.1016/S0076-6879(10)75013-8 PMC3205452

[b23] Manosas M., Xi X. G., Bensimon D. & Croquette V. Active and passive mechanisms of helicases. Nucleic Acids Res. 38, 5518–5526 (2010). 20423906 10.1093/nar/gkq273 PMC2938219

[b24] Dessinges M. N., Lionnet T., Xi X. G., Bensimon D. & Croquette V. Single-molecule assay reveals strand switching and enhanced processivity of UvrD. Proc. Natl Acad. Sci. USA 101, 6439–6444 (2004). 15079074 10.1073/pnas.0306713101 PMC404063

[b25] Dumont S. *et al.* RNA translocation and unwinding mechanism of HCV NS3 helicase and its coordination by ATP. Nature 439, 105–108 (2006). 16397502 10.1038/nature04331 PMC1560093

[b26] Morris P. D. & Raney K. D. DNA helicases displace streptavidin from biotin-labeled oligonucleotides. Biochemistry 38, 5164–5171 (1999). 10213622 10.1021/bi9822269

[b27] Giedroc D. P., Khan R. & Barnhart K. Overexpression, purification, and characterization of recombinant T4 gene 32 protein22-301 (g32P-B). J. Biol. Chem. 265, 11444–11455 (1990). 2195020

[b28] Hatch K., Danilowicz C., Coljee V. & Prentiss M. Direct measurements of the stabilization of single-stranded DNA under tension by single-stranded binding proteins. Phys. Rev. E Stat. Nonlin. Soft. Matter Phys. 76, 021916 (2007). 17930074 10.1103/PhysRevE.76.021916

[b29] Qu X., Lancaster L., Noller H. F., Bustamante C. & Tinoco I. Jr. Ribosomal protein S1 unwinds double-stranded RNA in multiple steps. Proc. Natl Acad. Sci. USA 109, 14458–14463 (2012). 22908248 10.1073/pnas.1208950109 PMC3437903

[b30] Linder P. & Fuller-Pace F. V. Looking back on the birth of DEAD-box RNA helicases. Biochim. Biophys. Acta 1829, 750–755 (2013). 23542735 10.1016/j.bbagrm.2013.03.007

[b31] Pyle A. M. Translocation and unwinding mechanisms of RNA and DNA helicases. Annu. Rev. Biophys. 37, 317–336 (2008). 18573084 10.1146/annurev.biophys.37.032807.125908

[b32] Myong S. *et al.* Cytosolic viral sensor RIG-I is a 5'-triphosphate-dependent translocase on double-stranded RNA. Science 323, 1070–1074 (2009). 19119185 10.1126/science.1168352 PMC3567915

[b33] Lee K. S., Balci H., Jia H., Lohman T. M. & Ha T. Direct imaging of single UvrD helicase dynamics on long single-stranded DNA. Nat. Commun. 4, 1878 (2013). 23695672 10.1038/ncomms2882 PMC3674262

[b34] Beran R. K., Bruno M. M., Bowers H. A., Jankowsky E. & Pyle A. M. Robust translocation along a molecular monorail: the NS3 helicase from hepatitis C virus traverses unusually large disruptions in its track. J. Mol. Biol. 358, 974–982 (2006). 16569413 10.1016/j.jmb.2006.02.078

[b35] Cheng W., Dumont S., Tinoco I. Jr. & Bustamante C. NS3 helicase actively separates RNA strands and senses sequence barriers ahead of the opening fork. Proc. Natl Acad. Sci. USA 104, 13954–13959 (2007). 17709749 10.1073/pnas.0702315104 PMC1955789

[b36] Betterton M. D. & Julicher F. Opening of nucleic-acid double strands by helicases: active versus passive opening. Phys. Rev. E Stat. Nonlin. Soft. Matter Phys. 71, 011904 (2005). 15697627 10.1103/PhysRevE.71.011904

[b37] Soultanas P., Dillingham M. S., Wiley P., Webb M. R. & Wigley D. B. Uncoupling DNA translocation and helicase activity in PcrA: direct evidence for an active mechanism. EMBO J. 19, 3799–3810 (2000). 10899133 10.1093/emboj/19.14.3799 PMC313991

[b38] Velankar S. S., Soultanas P., Dillingham M. S., Subramanya H. S. & Wigley D. B. Crystal structures of complexes of PcrA DNA helicase with a DNA substrate indicate an inchworm mechanism. Cell 97, 75–84 (1999). 10199404 10.1016/s0092-8674(00)80716-3

[b39] Dillingham M. S., Webb M. R. & Kowalczykowski S. C. Bipolar DNA translocation contributes to highly processive DNA unwinding by RecBCD enzyme. J. Biol. Chem. 280, 37069–37077 (2005). 16041061 10.1074/jbc.M505520200

[b40] Dillingham M. S. & Kowalczykowski S. C. RecBCD enzyme and the repair of double-stranded DNA breaks. Microbiol. Mol. Biol. Rev. 72, 642–671 (2008). 19052323 10.1128/MMBR.00020-08 PMC2593567

[b41] Honda M., Park J., Pugh R. A., Ha T. & Spies M. Single-molecule analysis reveals differential effect of ssDNA-binding proteins on DNA translocation by XPD helicase. Mol. Cell. 35, 694–703 (2009). 19748362 10.1016/j.molcel.2009.07.003 PMC2776038

[b42] Cordin O. & Beggs J. D. RNA helicases in splicing. RNA. Biol. 10, 83–95 (2013). 23229095 10.4161/rna.22547 PMC3590240

[b43] Parsyan A. *et al.* mRNA helicases: the tacticians of translational control. Nat. Rev. Mol. Cell. Biol. 12, 235–245 (2011). 21427765 10.1038/nrm3083

[b44] Le Hir H. & Andersen G. R. Structural insights into the exon junction complex. Curr. Opin. Struct. Biol. 18, 112–119 (2008). 18164611 10.1016/j.sbi.2007.11.002

[b45] Page M. F., Carr B., Anders K. R., Grimson A. & Anderson P. SMG-2 is a phosphorylated protein required for mRNA surveillance in *Caenorhabditis elegans* and related to Upf1p of yeast. Mol. Cell. Biol. 19, 5943–5951 (1999). 10454541 10.1128/mcb.19.9.5943 PMC84455

[b46] Gregersen L. H. *et al.* MOV10 Is a 5' to 3' RNA helicase contributing to UPF1 mRNA target degradation by translocation along 3' UTRs. Mol. Cell. 54, 573–585 (2014). 24726324 10.1016/j.molcel.2014.03.017

[b47] Hogg J. R. & Goff S. P. Upf1 senses 3'UTR length to potentiate mRNA decay. Cell 143, 379–389 (2010). 21029861 10.1016/j.cell.2010.10.005 PMC2981159

[b48] Hurt J. A., Robertson A. D. & Burge C. B. Global analyses of UPF1 binding and function reveal expanded scope of nonsense-mediated mRNA decay. Genome Res. 23, 1636–1650 (2013). 23766421 10.1101/gr.157354.113 PMC3787261

[b49] Kurosaki T. *et al.* A post-translational regulatory switch on UPF1 controls targeted mRNA degradation. Genes Dev. 28, 1900–1916 (2014). 25184677 10.1101/gad.245506.114 PMC4197951

[b50] Kurosaki T. & Maquat L. E. Rules that govern UPF1 binding to mRNA 3' UTRs. Proc. Natl Acad. Sci. USA 110, 3357–3362 (2013). 23404710 10.1073/pnas.1219908110 PMC3587222

[b51] Zund D., Gruber A. R., Zavolan M. & Muhlemann O. Translation-dependent displacement of UPF1 from coding sequences causes its enrichment in 3' UTRs. Nat. Struct. Mol. Biol. 20, 936–943 (2013). 23832275 10.1038/nsmb.2635

[b52] Melero R. *et al.* The cryo-EM structure of the UPF-EJC complex shows UPF1 poised toward the RNA 3' end. Nat. Struct. Mol. Biol. 19, 498–505 (2012). 22522823 10.1038/nsmb.2287

[b53] Muller-McNicoll M. & Neugebauer K. M. How cells get the message: dynamic assembly and function of mRNA-protein complexes. Nat. Rev. Genet. 14, 275–287 (2013). 23478349 10.1038/nrg3434

[b54] Muhlemann O. & Jensen T. H. mRNP quality control goes regulatory. Trends Genet. 28, 70–77 (2012). 22154474 10.1016/j.tig.2011.11.001

[b55] Nelson S. W., Kumar R. & Benkovic S. J. RNA primer handoff in Bacteriophage T4 DNA replication—the role of single-stranded DNA binding protein and polymerase accessory proteins. J. Biol. Chem. 283, 22838–22846 (2008). 18511422 10.1074/jbc.M802762200 PMC2504890

[b56] Manosas M., Perumal S. K., Croquette V. & Benkovic S. J. Direct observation of stalled fork restart via fork regression in the T4 replication system. Science 338, 1217–1220 (2012). 23197534 10.1126/science.1225437 PMC3858903

[b57] Walmacq C., Rahmouni A. P. & Boudvillain M. Influence of substrate composition on the helicase activity of transcription termination factor Rho: reduced processivity of Rho hexamers during unwinding of RNA-DNA hybrid regions. J. Mol. Biol. 342, 403–420 (2004). 15327943 10.1016/j.jmb.2004.07.026

[b58] Chase J. W. & Williams K. R. Single-stranded DNA binding proteins required for DNA replication. Annu. Rev. Biochem. 55, 103–136 (1986). 3527040 10.1146/annurev.bi.55.070186.000535

[b59] Krauss G., Sindermann H., Schomburg U. & Maass G. Escherichia coli single-strand deoxyribonucleic acid binding protein: stability, specificity, and kinetics of complexes with oligonucleotides and deoxyribonucleic acid. Biochemistry 20, 5346–5352 (1981). 7028102 10.1021/bi00521a040

