# Supplementary material for: Human Upf1 is a highly processive RNA helicase and translocase with RNP remodelling activities
Source: Nat Commun. 2015 Jul 3;6:7581. doi: 10.1038/ncomms8581 (PMC4506499; doi:10.1038/ncomms8581)
Supplement: Supplementary Information — Supplementary Figures 1-10 and Supplementary Tables 1-3 [file ncomms8581-s1.pdf]

Supplementary material of: Human Upf1 is a highly processive RNA helicase and translocase with RNP remodelling activities

By Francesca Fiorini, Debjani Bagchi, Hervé Le Hir and Vincent Croquette

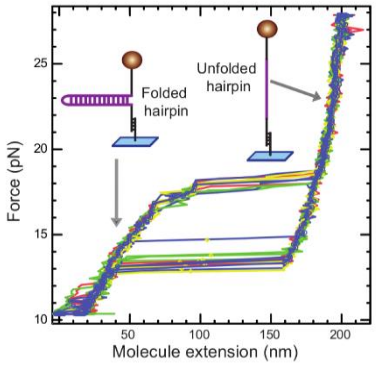

Supplementary Figure 1: Mechanical properties of RNA hairpin. Force-extension curve for the RNA hairpin (156 bp) demonstrating the stable hairpin folding below ~17 pN of force applied. At this threshold, the extension abruptly increased showing the mechanical unfolding of the hairpin. On lowering the applied force to ~13 pN, the RNA rapidly refolded and returned to its initial extension

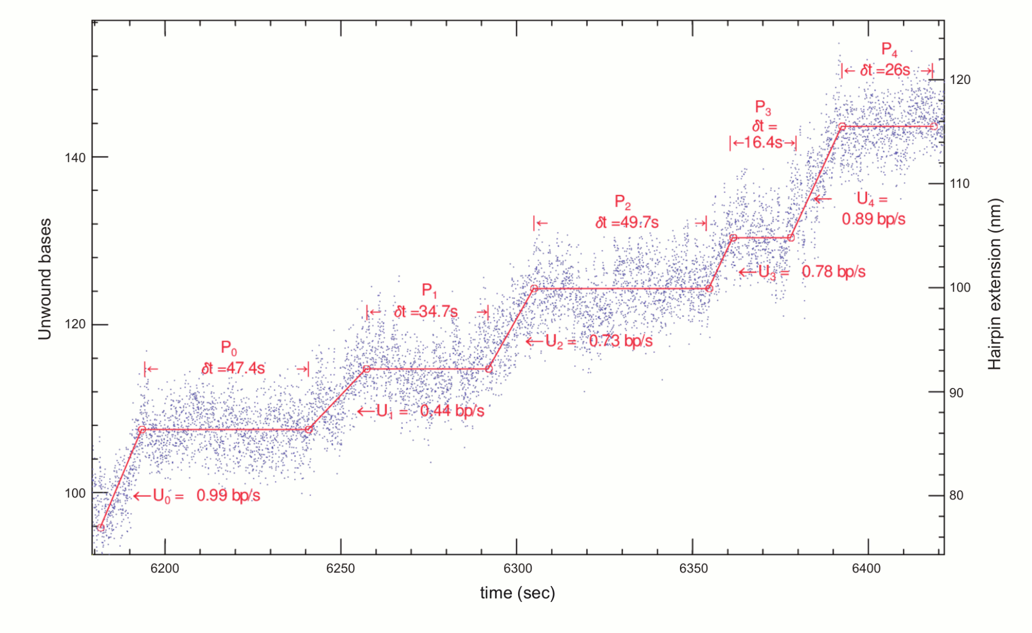

Supplementary Figure 2: The unwinding event of Fig. 1c is zoomed at higher resolution, illustrating the complex motion of Upf1-HD on the RNA hairpin. The enzyme alternates short phases of unwinding (U_i) separated by pauses (P_i). The pause durations are random in time. The unwinding short events are also random both in duration and in their rate. The enzyme traces have been analysed using this pattern of events. While isolating unwinding events and pauses is relatively easy when their duration is long, it is more delicate when these events becomes short and noise dominates. This limits the analysis of short events

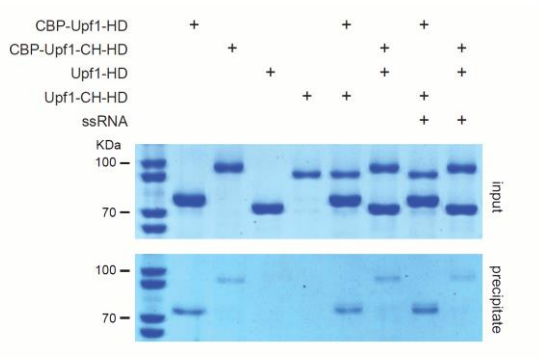

Supplementary Figure 3: Upf1 does not dimerize Co-precipitations with CBP-UPF1-HD or CBP-UPF1-CH-HD mixed with UPF1-HD or UPF1-CH-HD with or without ssRNA, after incubation under binding conditions. Protein mixtures before (input, 20% of total) or after precipitation (precipitate) were separated by SDS-PAGE on 10% (w/v) acrylamide gels.

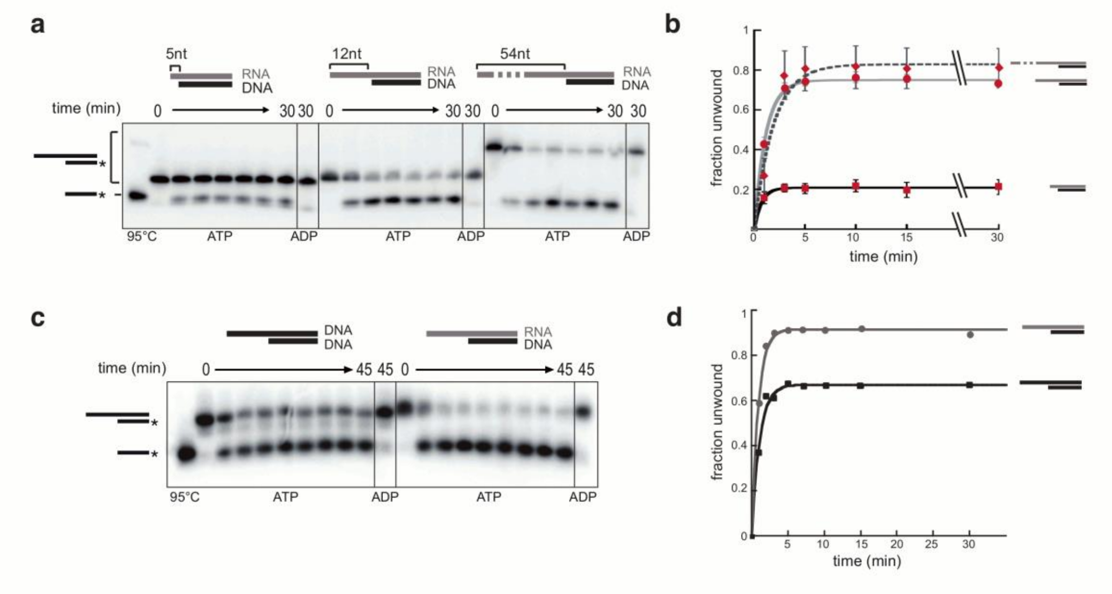

Supplementary Figure 4: Upf1 unwinds NA hybrids in a non-cooperative manner

(**a**) Unwinding assay using Upf1-HD and dsNA substrates with 5' overhangs of 5, 12 and 54 nts (depicted on the top). The Upf1-HD must bind ~10 nts of ssRNA[13] before unwinding. (**b**) Graph showing the fraction of DNA oligonucleotide released over time in the presence of Upf1-HD. Data points derived from three independent experiments were fitted using Kaleidagraph (Synergy software) to the pseudo-first order equation $y=A[1-e^{(-kt)}]$, where A and k represent, respectively, the amplitude and the rate constant of the unwinding reaction[57]. The unwinding rate observed for the different substrates was independent of the length of the ssRNA overhang, indicating that Upf1 activity is non-cooperative. (**c**) A representative time-course experiment of dsDNA and RNA/DNA duplexes (depicted on the top) unwound by Upf1-HD. In control lanes ATP was replaced with ADP or the sample was heat-denatured (95°C). (**d**) Graph showing the fraction of DNA oligonucleotide released over time in the presence of Upf1-HD.

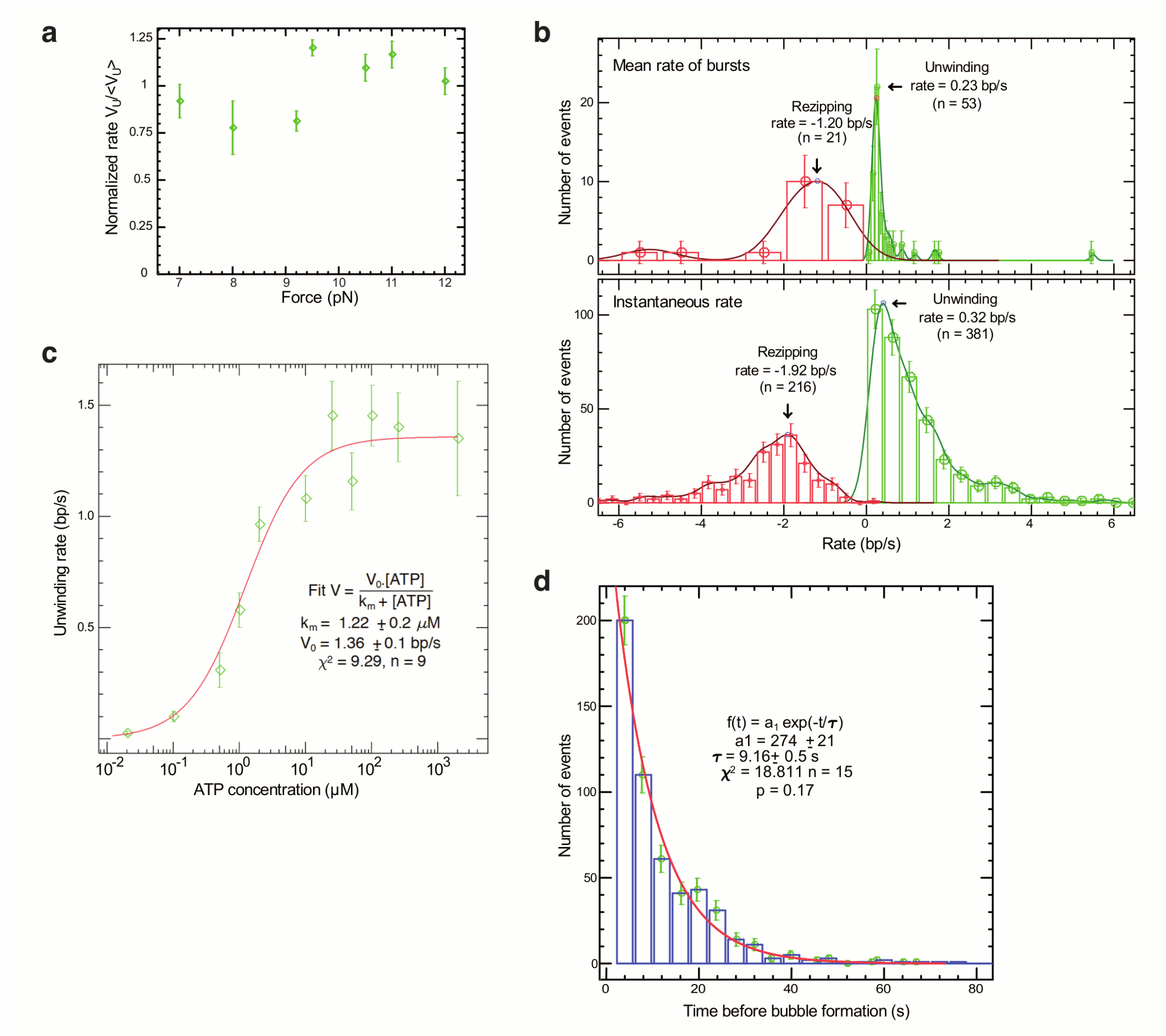

Supplementary Figure 5: Measurement of unwinding and translocation velocity of Upf1. (**a**) Graph of mean unwinding velocities (V_{Un}) measured as a function of the pulling force. The unwinding speed of Upf1-HD is mostly constant for a range of forces between 7–12 pN that is applied to the magnetic bead (and hence to the hairpin fork). (**b**) Distributions of unwinding and translocation rates of Upf1-HD on a DNA substrate. (**c**) Unwinding velocities of Upf1-HD expressed as a function of ATP concentration. The data were fitted to a first order Michaelis-Menten kinetics ($V = [ATP]\ V_{max} / ([ATP] + K_M)$) where V_{max} = 1.36 ± 0.1 bp/s and in a K_M of 1.22 ± 0.2 µM obtained with a χ^2= 9.29. (**d**) The probability distribution of the time taken to form the bubble in the hairpin after the force is lowered to nearly 4.6 pN. This bubble-initiation time t_{bubble} is exponentially distributed, with a mean τ = 9.16 ± 0.5 sec.

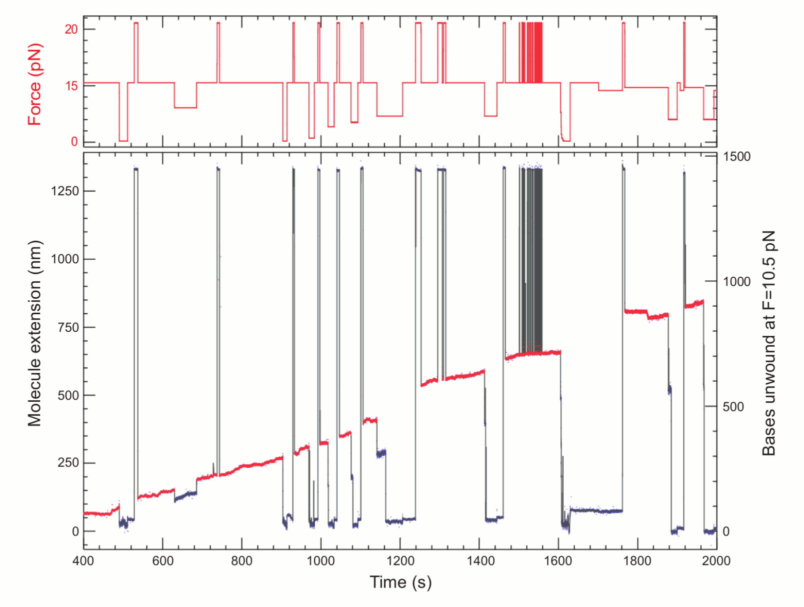

Supplementary Figure 6: Evolution of the position of a Upf1-HD helicase unwinding a 1.2 kb DNA hairpin, extended trace of Fig. 2 d. During the process, the force applied to the hairpin is modulated as shown on the top trace. When the force reaches 20pN, the hairpin is mechanically opened. Most of the time the force is held at 10.5 pN showing the action of the helicase. When the force is decreased below 5pN the hairpin refolds entrapping the Upf1-HD which remains bound to the DNA. When the force is increased back to 10.5 pN the hairpin remains closed, when a force surge to 20pN is applied briefly the hairpin resumed is previous open position increased by a small amount corresponding to the travel of the helicase in the bubble. Notice that the enzyme has travelled steadily along the NA for more than 1600s

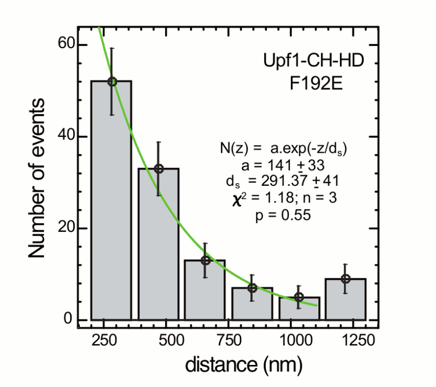

Supplementary Figure 7 Processivity of Upf1^{F192E} in the re-zipping mode. Distribution of the distance travelled by Upf1^{F192E} while re-zipping a hairpin before a strand switching occurrence. This processivity is considerably reduced compared with the processivity of the enzyme in the unwinding mode which exceeds 16kb.

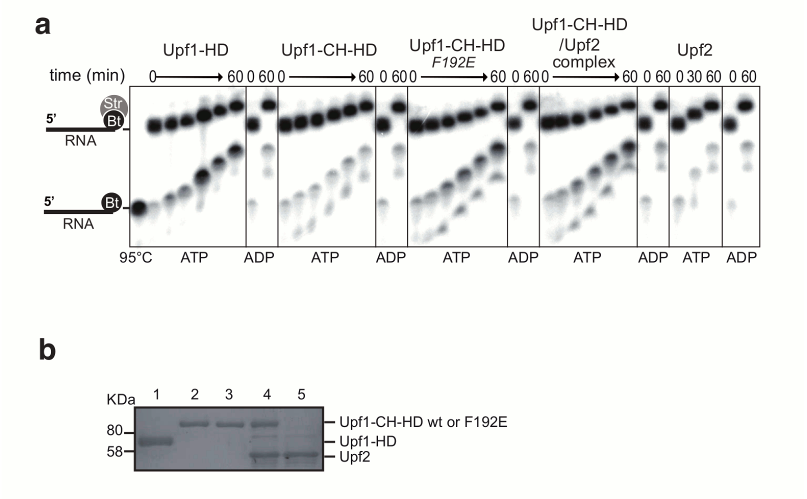

Supplementary Figure 8 Active translocating Upf1 disrupts biotin-streptavidin interactions. (**a**) Time course of an active displacement of streptavidin from a 3' biotinylated RNA by Upf1-HD, Upf1-CH-HD, Upf1^{F192E}, and Upf1-CH-HD/Upf2. In a control experiment ADP replaced the ATP. At various times (see Methods section) aliquots from the helicase reaction were mixed in a quench buffer and loaded immediately on 8% polyacrylamide gel containing 0.3% of SDS to selectively denature Upf1-RNA complexes. (**b**) SDS–PAGE showing the Upf1-HD, Upf1, Upf1^{F192E}, and Upf1-CH-HD/Upf2 proteins used for displacement assay.

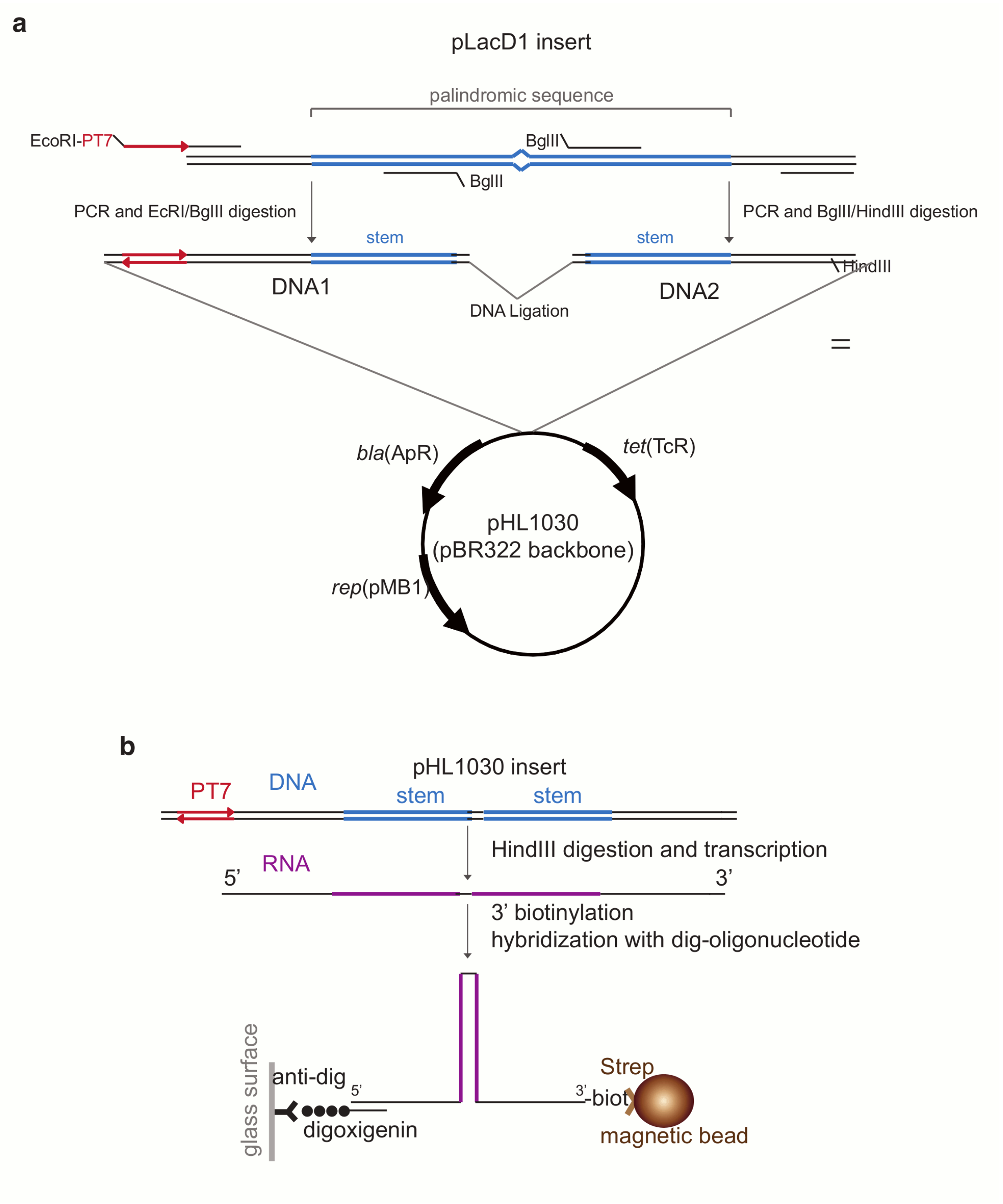

Supplementary Figure 9: Schematic representation of pHL1030 construction (see Material and methods).

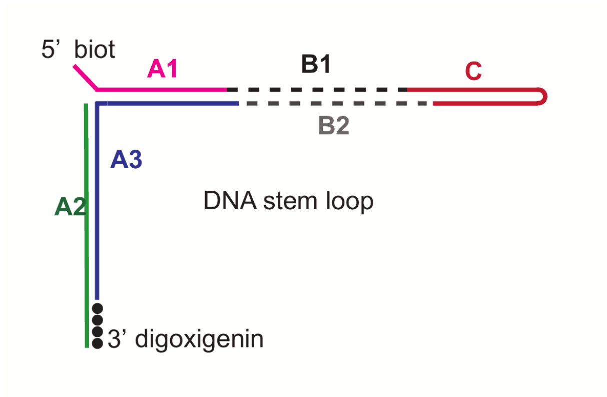

Supplementary Figure 10: DNA and RNA hairpin constructions. (**a**) Schematic representation of the DNA hairpin (1.2 Kbp) substrates construction. (**b**) RNA hairpin (156 bp) production (see Material and methods).

Supplementary Table 1

	**Upf1-HD**	**Upf1-HD**
Substrate	RNA	DNA
Unwound	2.85 kb	35.4 kb
Duration	4 hours	60.9 hours
**Mean rate**	**0.19 bp/s**	**0.162 bp/s**
τ_u	11.7 ± 3 s (n = 50)	12.8 ± 2s (n = 151)
V_u	0.59 bp/s (n = 92)	0.32 bp/s (n = 381)
τ_{PU}	13.5 ± 4 s (n = 53)	20 ± 3 s (n = 150)
τ_T	5.5 ± 2s (n = 35)	3.9 ± 1s (n = 115)
V_T	0.79 bp/s (n = 41)	1.92 bp/s (n = 216)
τ_{PT}	2.4 ± 1.5s (n = 36)	4.2 ± 1s (n = 152)
Processivity factor (on 1.2kb)		52/56

Supplementary Table 1: Comparison of Upf1-HD rates on RNA and DNA substrates. The mean rate corresponds to the rate in a bulk experiment divided by the number of bases unwound in the duration of the activity. τ_U characterizes the mean duration of unwinding phases occurring at rate V_u, followed by a pause lasting typically τ_{PU}. Similarly, translocating (re-zipping) bursts are defined as translocating phases at rate V_T lasting typically τ_T interrupted by pauses lasting τ_{PT}. The total number of measurements, *n*, used for the calculations, is indicated.

Supplementary Table 2

	**Upf1-CH-HD**	**Upf1-CH-HD/Upf2**	**Upf1F192E**
Substrate	DNA	DNA	DNA
Activity	49%	81%	99.1%
Unwound	32 kb	31.7 kb	117.5 kb
Duration	70.5 hours	58.6 hours	28.5 hours
**Mean rate**	**0.126 bp/s**	**0.150 bp/s**	**1.145 bp/s**
τ_u	N.A.	6.7 ± 1 s (n = 139)	4.19 ± 0.5 s (n= 192)
Vu	N.A.	0.53 ± 0.15 bp/s (n =475)	1.77 ± 0.25 bp/s (n =672)
τ_{PU}	N.A.	7.5 ± 1 s (n = 98)	4.4 ± 0.5 s (n =158)
τ_T	N.A.	N.A.	3 ± 0.35 s (n = 162)
V_T	N.A.	N.A.	-2.57 ± 0.33 bp/s (n =431)
τ_{PT}	N.A.	N.A.	2.5 ± 0.3 s (n = 167)
Processivity factor (on 1.2kb)		24/25	55/56

Supplementary Table 2: Comparison of Upf1-CH-HD, Upf1-CH-HD/Upf2 and Upf1^{F192E}**.** The mean rate is equivalent to the rate in a bulk experiment, and it is obtained by dividing the number of bases unwound, by the time duration of the activity. τ_U characterizes the mean duration of unwinding phases occurring at rate V_U, followed by a pause lasting typically $\tau_{PU.}$ Similarly, translocating (re-zipping) bursts are defined as translocating phases at rate V_T lasting typically τ_T interrupted by pauses lasting τ_{PT}. For Upf1-CH-HD, the unwinding traces are mostly blocked and the statistics of unwinding traces is insufficient to measure τ_U and τ_{PU}. For Upf1-CH-HD/Upf2, there is not enough translocation statistics to measure the characteristic parameters. The total number of measurements, *n*, used for the calculations, is indicated.

Supplementary Table 3

Oligonucleotide	*Sequence*
A1	5'BiotinTTTTTTTTTTTTTTTTTTTTTTTTTTTTTTTTTTTTTTTTTTTTTTTTTTGGATTCGCGGGTCTCT
A2	AACCGTCCTTTACTTGTCATGCGCTCTAATCTCTGGGCATCTGGCTATGATGTTGATGGAACTGACC AAACGTCGGTGGG
A3	AGGAAGAGACCCGCGAATCCCCCACCGACGTTTGGTCAGTT
C	TCGCGCCTGATCGTCCACTTTTTTTAGTGGACGATCAGGC
B1-AT	TCCTATTTAAATATTAATATTTTTATAATTTTAAATATATAAATAATAATAATTAAAAAATTAATATTTTTTGTTTTATAATATATAATAATTTAAAAAAAATTTAAAATTTAAATATAAATTAAATATTTTATTAATA
B2-AT	TATTAATAAAATATTTAATTTATATTTAAATTTTAAATTTTTTTTAAATTATTATATATTATAAAACAAAAAATATTAATTTTTTAATTATTATTATTTATATATTTAAAATTATAAAAATATTAATATTTAAAT
HLH1853	CAGAATTCCTAATACGACTCACTATAGGGAGCTCACTCATTAGGCAC
HLH2124	CATGACAGATCTCATTACGTCTTAACTCTTCATATTTAG
HLH2125	CATGACAAGCTTGCCATTCAGGCTGCGCAACTGTTGGG
HLH2181	GTAAAGCCTGGGGTGCCTAATGAGTGAGCTCCC-4Xdigoxigenin
HLH1172	GGTACCCGCAGCTTCTCGAGG
HLH1164	CGAAATTAATACGACTCACTATAGGG
HLH1376	AATTAATACGACTCACTATAGGGATCCTCGAGAAGCTGCGGGTACC
HLH1377	AATTAATACGACTCACTATAGGGGATCTGGATCCTCGAGAAGCTGCGGGTACC
HLH2653	CCATGGGCGTCGAAGAGCTCCTAGGTCTAGACTATGGTAGCTATTTAAGTTTGAGTTATGGCGAGTAGGCCAGGGATATCACTCAGCATAATTAA
